# Impact of shorter picking intervals on the storability and postharvest quality of rabbiteye blueberries cv. ‘Brightwell’

**DOI:** 10.3389/fpls.2025.1683940

**Published:** 2025-10-17

**Authors:** Amit Godara, Zilfina Rubio Ames, Angelos Deltsidis

**Affiliations:** Department of Horticulture Sciences, University of Georgia, Tifton, GA, United States

**Keywords:** maturity, cold storage, delayed harvest, firmness, weight loss, total soluble solids, anthocyanins, titratable acidity

## Abstract

The quality and shelf-life of fresh-market blueberries are crucial aspects for both growers and consumers. Different picking intervals could be affecting these factors, and understanding changes associated with these issues is essential to optimize postharvest fruit performance. This study evaluated the impacts of different picking intervals on the postharvest quality and storability of rabbiteye blueberries (*Vaccinium virgatum*) cv. ‘Brightwell’ in Georgia, USA, during the 2023 and 2024 seasons. Harvesting was carried out at intervals of two days (Trt A), three days (Trt B), and seven days (Trt C), with three harvests per treatment. The main quality parameters assessed included berry damage (%), berry diameter, weight loss, firmness, total soluble solids, titratable acidity, and total anthocyanin concentration, measured over 21 days of storage at 1 °C and 85% relative humidity. Results demonstrated that fruit harvested with the Trt C (seven days interval) exhibited significantly higher weight loss of up to 15.5% at 21 days after storage in 2024, greater berry damage (ranging from 27% to 41.5%), and lower firmness (151.6-155.0 g·mm^−1^ at harvest 3) than shorter harvesting intervals treatments. Conversely, the 7-day interval yielded higher total soluble solids at harvest 3 (14.0%) versus 2- and 3-day intervals (12.5-13.2%), lower titratable acidity than the 2-day interval (Trt A highest at 1.51-1.53% at Harvest 3), and the highest total anthocyanins (Trt C: 258.9-267.2 mg·L^−1^). Frequent harvesting (Trt A and B) helped maintain higher fruit firmness, reduced weight loss, and minimized postharvest berry damage while maintaining optimal sugars and acid levels. These findings highlight the importance of optimizing picking intervals, indicating that a three-day picking interval (Trt B) is an effective option for maintaining postharvest fruit quality and storage potential for fresh market blueberries. The 7-day interval (Trt C) produced fruit with higher anthocyanin content, total soluble solids, and lower firmness, indicating greater suitability for processing rather than fresh market use. This study provides valuable insights for blueberry growers aiming to improve the postharvest life of rabbiteye blueberries under warm and humid climate conditions.

## 1 Introduction

Blueberries (*Vaccinium* spp.), native to North America, are now cultivated in approximately 27 countries worldwide. The United States is considered the largest blueberry producer globally, yielding around 294 thousand metric tons from 41,683 harvested hectares in 2023 (US Department of Agriculture, 2024; USHBC, 2024). In recent years, consumer demand and scientific interest in this fruit have grown, particularly due to its nutritional value and antioxidant properties. Maintaining fruit quality from harvest to the consumer is essential for ensuring marketability and reducing postharvest losses (Chen et al., 2015; Edger et al., 2022; Evans and Ballen, 2014). Blueberry fruit development occurs in three stages: Stage I involves rapid cell division after fruit set; Stage II is a lag phase focused on seed maturation with minimal size change; and Stage III resumes growth through cell expansion, leading into ripening (Darnell et al., 1992; Godoy et al., 2008; Retamales, 2012). Ripening involves significant changes in fruit biochemical and metabolite profiles. This stage III marks the attainment of horticultural maturity, characterized by optimal sensory quality, including cell wall degradation, texture softening, modulation of organic acids, and elevated levels of soluble sugars and aroma volatiles (Erkan and Dogan, 2019). Visible changes that occur during the later stages of ripening are minimal (Giacalone et al., 2000). However, in this period, there are shifts in color, berry size, and internal fruit quality parameters such as total soluble solids (TSS) and titratable acidity (TA). For instance, as the fruit transitions from unripe to fully ripe, TSS increases while TA decreases (Eichholz et al., 2015; Sargent et al., 2006). Additionally, glucose and fructose are the primary sugars present in blueberries, and citric acid is the predominant organic acid, both of which contribute to the flavor profile of the fruit (Forney et al., 2010).

Blueberries exhibit ripening asynchrony, meaning fruit within the same cluster or on the same plant ripen at different times (Daviet et al., 2023; Vander Kloet and Cabilio, 2010). The degree of synchrony is influenced by both genetic factors, such as the inheritance of ripening uniformity and its relationship to crop load and environmental or management conditions, including temperature, pollination, production systems, and management practices (Lang and Danka, 1991; Luby and Finn, 1987; Nesmith, 2012; Ogden and van Iersel, 2009). Physiologically, asynchrony reflects variation in regulatory processes involving ethylene and abscisic acid (ABA) signaling, sugar and anthocyanin accumulation, and cell wall remodeling (Acharya et al., 2024; Ban et al., 2007; Wang et al., 2018; Zifkin et al., 2012). At the molecular level, differential expression of ripening-related genes, including those governing ethylene metabolism and anthocyanin biosynthesis (e.g., VcACS1, VcACO6, VcMYBA, VcUFGT), contributes to species and cultivar specific variation in ripening patterns (Chung et al., 2019; Li et al., 2024; Plunkett et al., 2018; Wang et al., 2022; Zifkin et al., 2012).

These genetic and physiological mechanisms underlying ripening asynchrony have direct implications for harvest management, as fruit within a single cluster may differ markedly in texture, size, and biochemical composition. Additionally, the sensory profile of blueberries remains relatively stable after harvest, which further emphasizes the importance of harvest timing and interval optimization to ensure consistent fruit quality during storage (Heidelbeere et al., 2018; Vander Kloet and Cabilio, 2010). The cuticular wax (“bloom”), which is present on the surface of the fruit, varies by cultivar and increases during ripening, playing a vital role in color appearance and postharvest quality (Chu et al., 2018; Yang, 2018). For Georgia growers, the primary sign of blueberry maturity is their color, with the berries generally deemed ready for harvest when they turn completely blue. However, despite their uniform appearance, blueberries at 100% blue stage within a cluster can vary in maturity stages and physiological age, with some being ripe and others overripe (Godara et al., 2023; Lobos et al., 2018; Moggia et al., 2017b). Therefore, surface color alone may no longer be a reliable indicator of physiological maturity (Lobos et al., 2018). The maturity stages of berries at harvest significantly affect the storage potential, as berries with an advanced maturity stage can result in softening and decay during storage (Lobos et al., 2018; Moggia et al., 2018). Blueberry growers are shifting to machine harvesting due to high labor demands and costs. This change, driven by labor shortages, has led to longer picking intervals to reduce yield loss associated with the harvest of unripe (green) fruit during frequent machine harvesting, often resulting in a higher percentage of overripe fruit being harvested (Gallardo et al., 2018; Lobos et al., 2018; Olmstead and Finn, 2014). Reducing the number of harvests by increasing the interval between successive picks can help reduce labor costs but may also negatively impact fruit quality, leading to higher postharvest losses (Galinato et al., 2016; Lyrene, 2006; Takeda et al., 2008). Early harvesting, particularly in hand-picked operations, may lead to firmer fruit with better shelf-life (Bremer et al., 2008). Additionally, mechanical harvesting tends to be performed at a more advanced maturity stage to maximize picking efficiency, which can result in greater postharvest losses due to reduced firmness and subsequent fruit damage (Olmstead and Finn, 2014). In regions like Georgia, where climatic conditions such as high temperatures and rainfall occur during harvesting season, extending the picking interval can result in a higher percentage of overripe berries, leading to increased weight loss and fruit softening during storage. Furthermore, it can increase issues such as fruit splitting, wet stem scar, sunburn, and loss of firmness, ultimately reducing the storage life and marketability of the fruit (Lobos et al., 2014; Lyrene, 2006; Marshall et al., 2006; Yang, 2018).

This study aims to evaluate the effects of different picking intervals on the postharvest quality of rabbiteye blueberries in Georgia, USA, focusing on key quality attributes such as berry diameter, firmness, weight loss, TSS, and TA over multiple storage durations. We hypothesize that longer harvest intervals will reduce berry firmness and increase postharvest weight loss due to the harvest of more overripe berries, thus decreasing marketable fruit postharvest. This research seeks to provide insights into optimizing picking intervals to minimize spoilage and enhance the overall quality and marketability of rabbiteye blueberries cv. ‘Brightwell.’

## 2 Materials and methods

### 2.1 Experimental site

**Figure 1:**
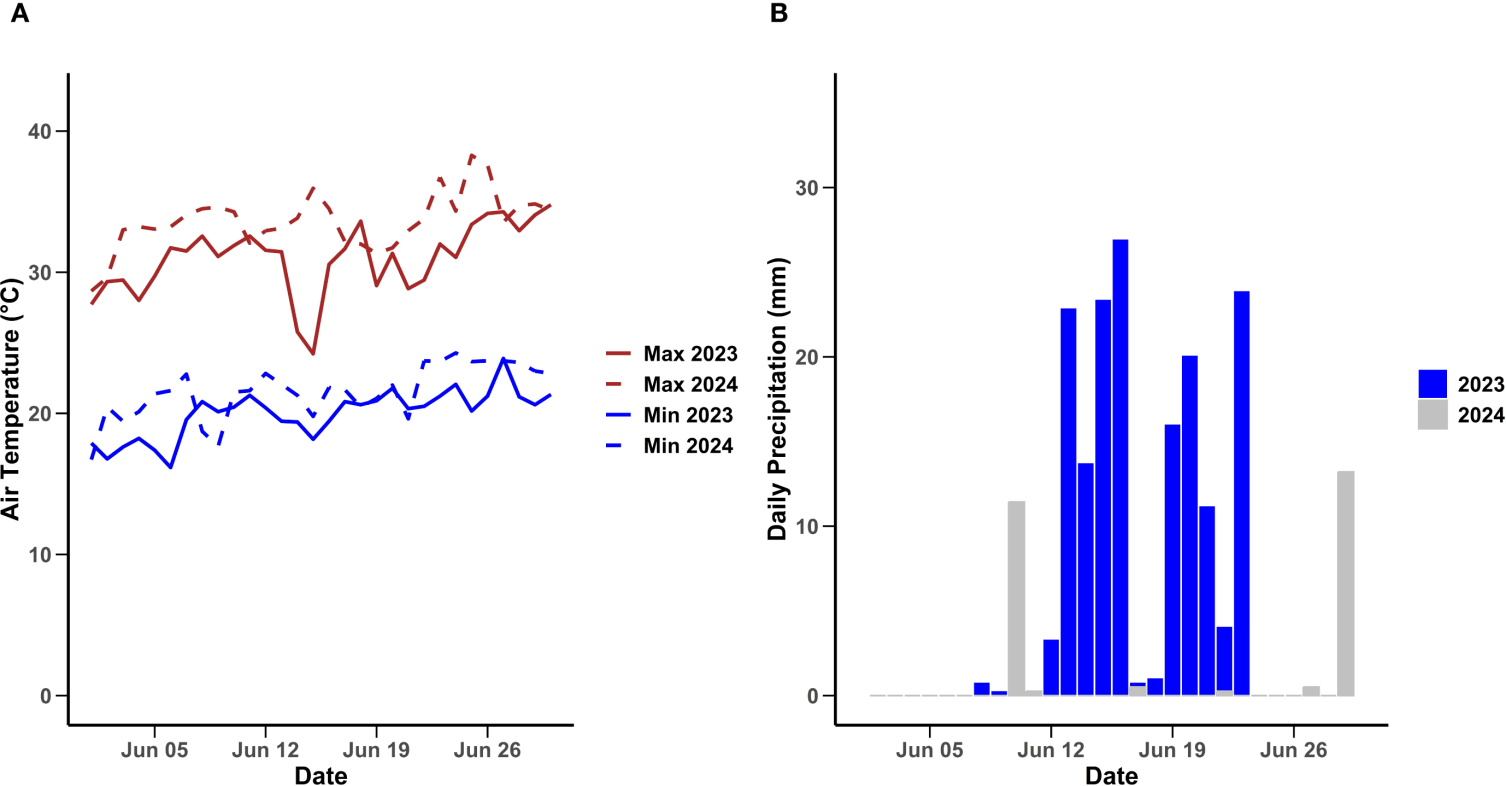
Maximum and minimum daily air temperature **(A)** and daily precipitation **(B)** in 2023 and 2024 from June 1 to June 31 at Blueberry Research Farm, Alma, Bacon County, GA. Weather data from the UGA Weather Network.

The field experiment on ‘Brightwell’ rabbiteye blueberries (*Vaccinium virgatum*) was conducted over the 2023 and 2024 seasons at the University of Georgia blueberry research farm in Alma, GA (lat. 31°32′05″N; long. 82°30′35″W). The cultivar Brightwell was selected for this study because it is a widely cultivated rabbiteye blueberry in Georgia and the Southeast, known for superior berry quality. The research site experiences a humid subtropical climate characterized by warm summers and frequent rainfall during harvest (**Figures 1A, B**). All agronomic practices, including fertilization, were conducted in accordance with the commercial blueberry guidelines established by the University of Georgia for blueberry production (Kissel and Sonon, 2018a, 2018).

### 2.2 Experimental design

**Figure 2:**
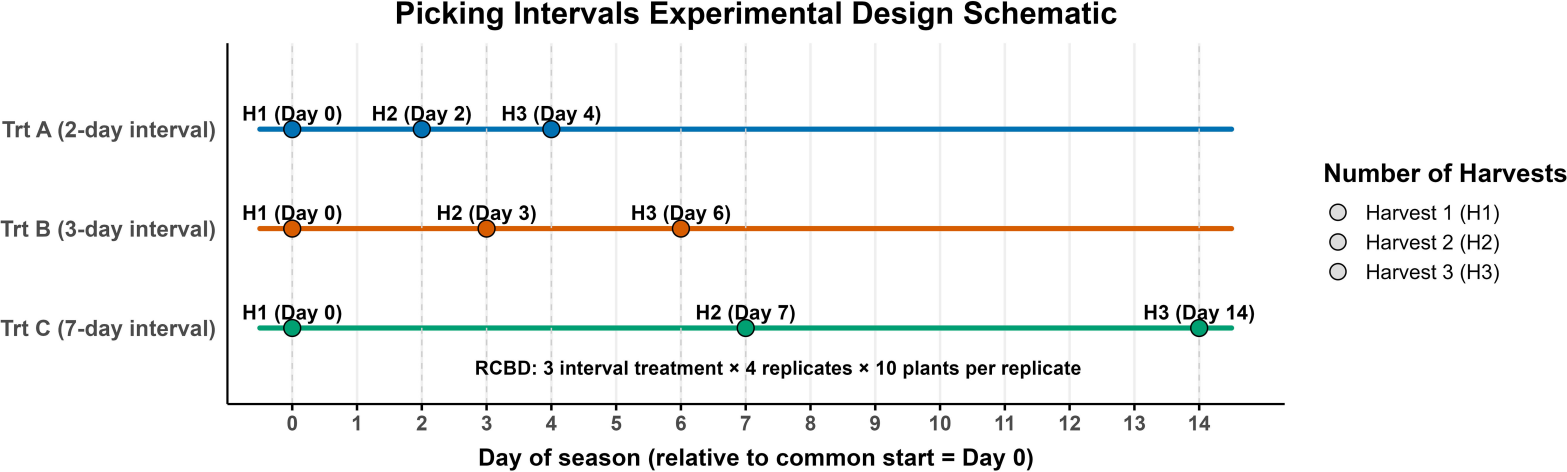
Experimental design for picking intervals. Three treatments were tested: Trt A (2-day), Trt B (3-day), and Trt C (7-day). Each treatment was harvested three times (H1-H3) on the following days (relative to Day 0): Trt A: 0, 2, 4; Trt B: 0, 3, 6; Trt C: 0, 7, 14. Circles indicate harvest events.

The experiment was established using a randomized complete block design with three picking intervals as the experimental factor: every two days (Trt A), every three days (Trt B), and every seven days (Trt C). Each treatment was replicated four times with 10 plants per replication. To simulate commercial harvesting conditions, the first harvest for all treatments was conducted on the same date, June 5 in 2023 and June 3 in 2024, corresponding to the beginning of the commercial harvest season. This common harvest date is referred to as Harvest 1, and it served as the starting point for each treatment’s specific harvest schedule. Harvest 2 and Harvest 3 represent the subsequent harvests for each treatment, based on their respective intervals. For example, Trt A (2-day interval) harvested on Day 0, Day 2, and Day 4; Trt B (3-day interval) on Day 0, Day 3, and Day 6; and Trt C (7-day interval) on Day 0, Day 7, and Day 14 (**Figure 2**).

Fruits were hand-harvested and stored in a airconditioned vehicle at ~19 °C during transportation to the Vidalia Onion Research Laboratory (Postharvest Lab) in Tifton, Georgia. Upon arrival, fruit were hand-sorted to retain only ripe berries, with small green berries removed and filled into vented 0.55 L clamshellls (one dry pint, Terra Box Florida LLC, Lakeland, FL) and stored at 1 °C and 85% relative humidity (RH) for up to 21 days. Fruit parameters were assessed at harvest and subsequently after 7, 14, and 21 days after storage (DAS). For each evaluation time point, four clamshells were used per replication, resulting in a total of 16 clamshells per evaluation (four replications × four clamshells). Since each harvest and treatment were evaluated four times during the study, a total of 64 clamshells (16 × 4) were used for quality assessments. Additionally, a separate set of clamshells was designated specifically for monitoring weight loss. These clamshells were weighed non-destructively at each storage evaluation time point (0, 7, 14, and 21 DAS), following the same storage conditions and arrangement. We hypothesized that longer picking intervals would lead to increased postharvest losses, particularly in firmness and weight loss, due to a greater proportion of overripe fruit being harvested.

### 2.3 Postharvest laboratory analysis

Weight loss was measured with a digital balance and calculated by subtracting the initial weight of the clamshell from the final weight of the clamshell containing fruit. Percentage (%) weight loss was calculated according to the following equation:


Weight Loss (%):(Wi−Wf)÷Wi×100

Where;

- W_i_ is the initial weight at harvest
- W_f_ is the final weight after 21 days of storage (21 DAS).

The postharvest quality traits were analyzed at harvest and subsequently weekly from the day of harvest (evaluation times of fruit were 7, 14, and 21 DAS, as indicated above). Berry damage, defined as splitting, juice leakage from the pedicel, wet scar, and skin tearing, was evaluated on 100 fruit samples per replicate. The Berry Damage percentage incidence was calculated as follows:


$$\text{Berry damage }\left(\%\right):\frac{\text{Number of oozing and splitting fruit}}{\text{Total number of tested fruit}}\times 100$$

Berry diameter and firmness were measured in 25 fruit per replication using a digital fruit firmness machine (FruitFirm^®^ 500, CVM Inc. Pleasanton, CA) equipped with a flat round compression plate (2.5 cm diameter). Firmness values are reported in the device’s original units (g·mm^−1^). The concentrations of total soluble solids (TSS), titratable acidity (TA), and total anthocyanins were determined using a 100 g aliquot of berries, homogenized with a tissue homogenizer (PowerGen 500, Fisher Scientific, Schwerte, Germany). The resulting slurry was centrifuged at 9,000 rpm at 4 °C (Sorvall X4R Pro-MD, Thermo Scientific, Osterode, Germany). The supernatant was filtered through cheesecloth, stored in plastic vials, and frozen at -20 °C for further analysis. TSS was measured by placing a small sample of blueberry supernatant on a digital refractometer (ATAGO, PAL-1, Model 3810, Japan), and the results were expressed as a percentage. For titratable acidity (TA), 6 mL of blueberry supernatant was diluted with 50 mL of deionized water and titrated to a pH of 8.2 using 0.1 mol L^−1^ NaOH with a titrator (916 Ti-Touch, Metrohm AG, Herisau, Switzerland). The TA was reported as a percentage of citric acid equivalents. Anthocyanin concentrations were measured according to the protocol described by Giusti and Wrolstad (2001). Briefly, blueberry supernatant was diluted separately with two different buffer solutions: a 0.025 M potassium chloride (KCl) buffer at pH 1.0, followed by a 0.4 M sodium acetate (CH_3_COONa) buffer at pH 4.5. Absorbance was measured using a microplate spectrophotometer, (BioTek, Epoch 2, Winooski, Vermont, USA) at two different wavelengths, 520 and 700 nm. A blank cell filled with deionized water was used as a reference. The monomeric anthocyanin concentration in the sample was calculated using the following formula:


$$\begin{array}{c} \text{Total Anthocyanin concentration }\left(\text{mg}\cdot {\text{L}}^{-1}\right):\\ \frac{A\times MW\times DF\times 1000}{\epsilon \times 1} \end{array}$$

Where;

- A (Absorbance at a given wavelength) = A= (A_520 nm_ - A_700 nm_) _pH 1.0_ – (A_520 nm_ - A_700 nm_) _pH 4.5_
- (A_520 nm_ - A_700 nm_) _pH 1.0_: Measures anthocyanin absorbance at pH 1.0
- (A_520 nm_ - A_700 nm_) _pH 4.5:_ Measures anthocyanin absorbance at pH 4.5
- MW: 449.2 (molecular weight of cyanidin-3-glucoside)
- DF: dilution factor
- Ɛ: 26,900 (molar absorptivity)

### 2.4 Statistical analysis

Data was subjected to analysis of variance (ANOVA), and one-way analysis of variance was conducted using JMP Pro 17 software (SAS Institute, Cary, NC) on variables measured at harvest and during postharvest storage. Normality was evaluated using Q-Q plots, and homogeneity of variances was confirmed using Levene’s test in JMP before conducting ANOVA. Analyses were conducted separately by year and by harvest. Comparisons were made between picking intervals at harvest and at each storage duration (7, 14, and 21 DAS), separately. The Tukey’s honestly significant difference (HSD) was used for mean separation at a significance level of α = 0.05. Graphs were generated using SigmaPlot 16.0 (Systat Software Inc., San Jose, CA) and RStudio software (RStudio, PBC, Vienna, Austria).

## 3 Results

Air temperatures in June of 2024 were elevated compared to June 2023 (**Figure 1A**), pointing to a warmer harvesting season. In contrast, precipitation levels throughout the month of June were greater in 2023 than in 2024 (**Figure 1B**). These findings highlight notable interannual fluctuations in both temperature and rainfall, which are essential for interpreting regional climate patterns.

### 3.1 Berry weight loss (%)

**Figure 3:**
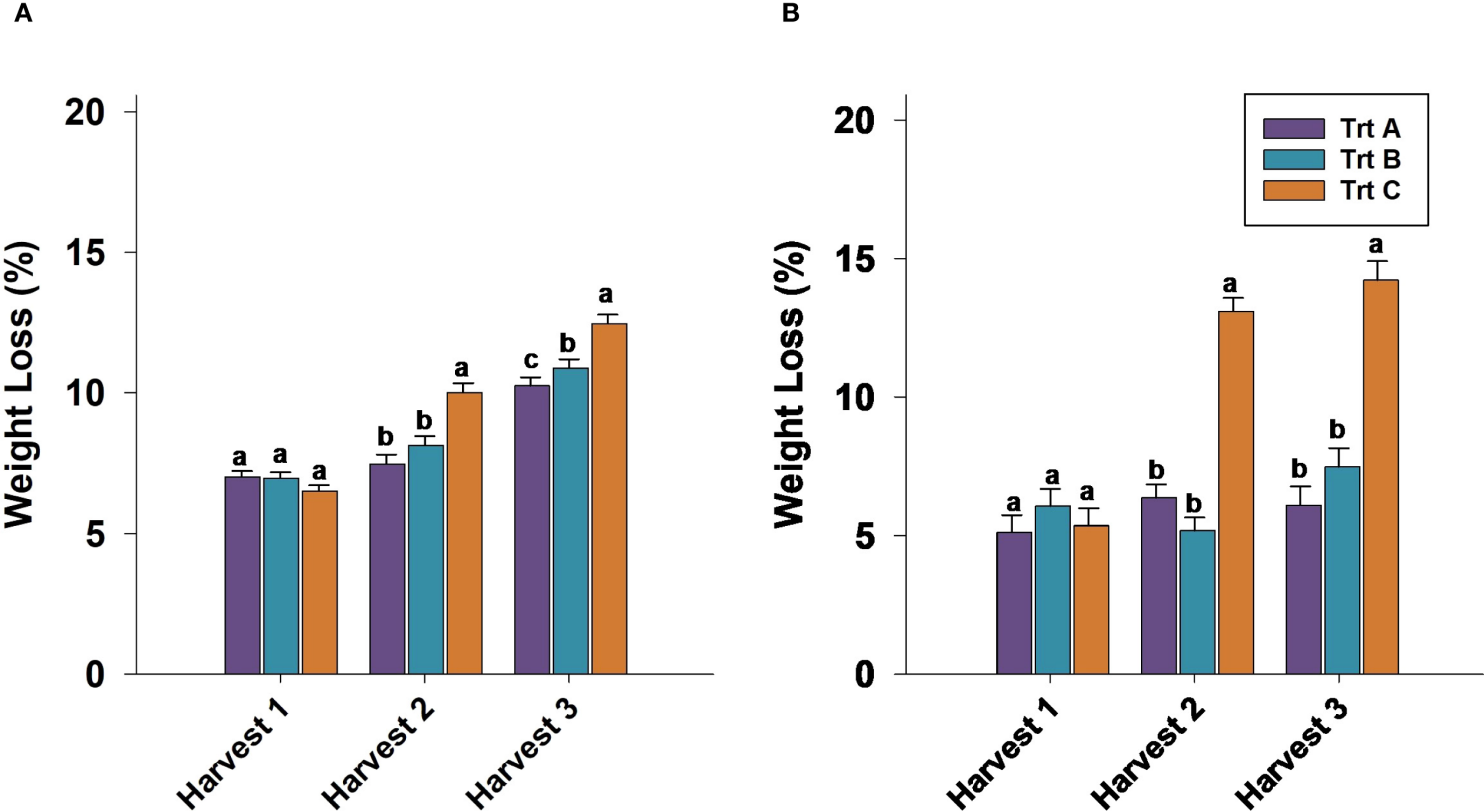
Effect of three different picking intervals on total weight loss (%) during 21 days of cold storage in 2023 **(A)** and 2024 **(B)**. Comparisons were made between treatments separately for each harvest. The means followed by the different letters are significantly different at *p* ≤ 0.05 based on Tukey’s honestly significant difference (HSD). Corresponding *p*-values are provided in **Supplementary Table 1**.

Berry weight was not significantly different between treatments after 21 days of cold storage in harvest 1 in 2023 and 2024. In 2023 and 2024, berries from Trt C (seven-day interval) consistently exhibited the highest weight loss compared to Trt A and B (two and three-day intervals, respectively) in harvests 2 and 3 (**Figures 3A, B**). Specifically, in 2024, 21 DAS weight loss for Trt C berries reached 15.5%, significantly higher than Trt A and B in harvest 3 (**Figure 3B**).

### 3.2 Berry damage (%)

**Figure 4:**
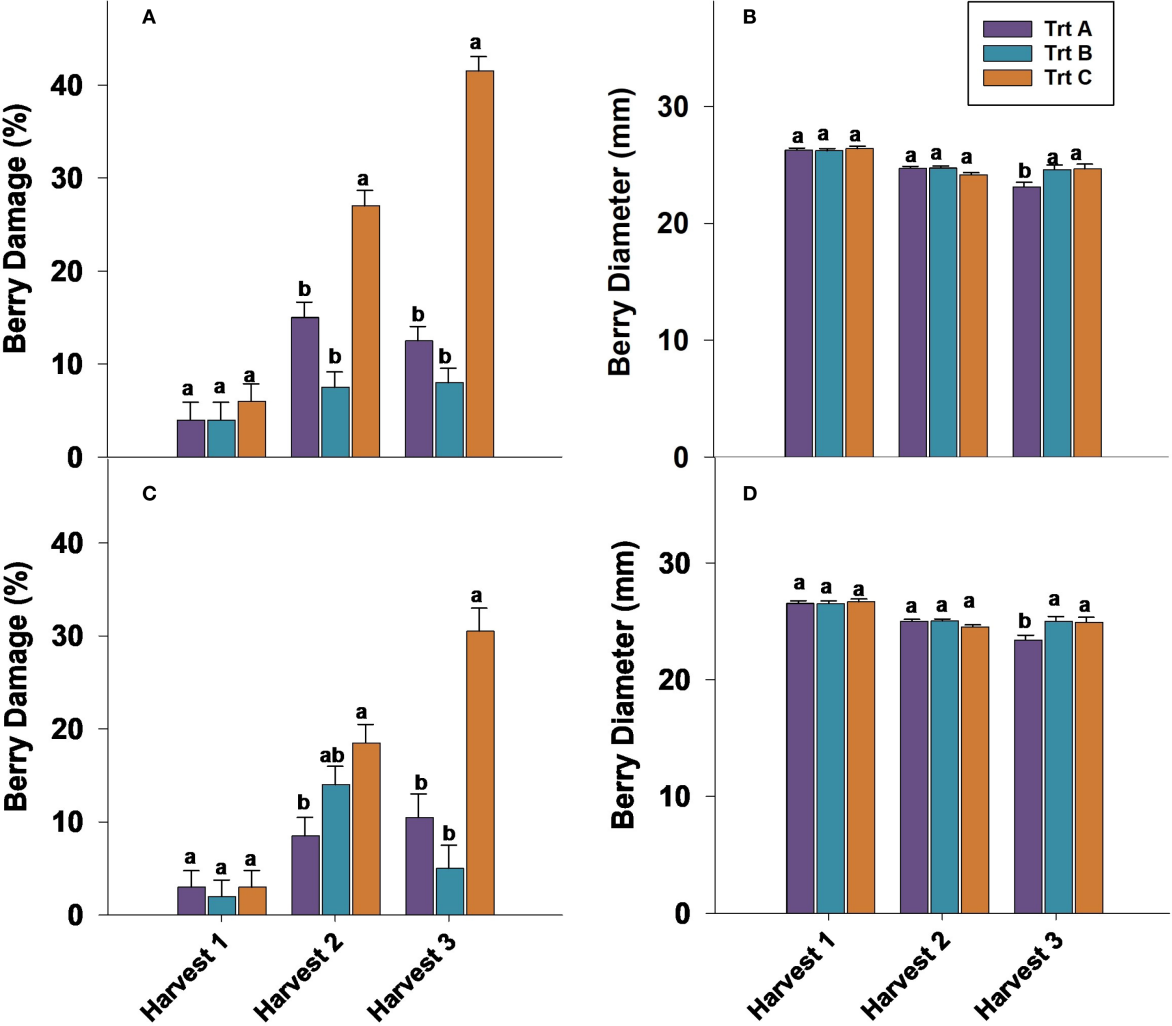
Effect of three different picking intervals on percentage of berry damage in 2023 **(A)** and in 2024 **(C)** and berry diameter in 2023 **(B)** and in 2024 **(D)** on ‘Brightwell’ cultivar at harvest. Comparisons were made between treatments separately for each harvest. The means followed by the different letters are significantly different at *p* ≤ 0.05 based on Tukey’s honestly significant difference (HSD). Corresponding *p*-values are provided in **Supplementary Table 1**.

The percentage of berry damage at harvest 1 showed no significant differences between treatments during the 2023 and 2024 seasons (**Figures 4A, C**). In 2023, Trt A and B had the lowest number of damaged berries at harvests 2 and 3, whereas Trt C exhibited the highest damage rates, with 27% and 41.5% of berries damaged at harvests 2 and 3, respectively (**Figure 4A**). In 2024, Trt C had a significantly higher percentage of damaged berries (30.5%) compared to Trt A at harvest 3 (**Figure 4C**). During storage, harvest 1 did not show significant differences in berry damage throughout storage duration (**Table 1**). In 2023, the damage percentage 21 DAS was 37.5% for harvest 2 and 44.5% for harvest 3. In 2024, these percentages were lower, at 20% for harvest 2 and 31% for harvest 3 (**Table 1**).

**Table 1:** Effects of picking intervals on fruit quality parameters of ‘Brightwell’ at each storage duration (7, 14, and 21 DAS) in 2023 and 2024.

Treatment^1^	Harvest	Storage day	Berry damage^2^ (%)		Berry diameter^2^ (mm)	
			2023	2024	2023	2024
Trt A	Harvest 1	7	7.5 ± 1.1 a	5.5 ± 1.1 a	25.8 ± 0.4 a	26.1 ± 0.4 a
Trt B	Harvest 1	7	10.5 ± 1.1 a	4.5 ± 1.1 a	25.3 ± 0.4 a	25.6 ± 0.4 a
Trt C	Harvest 1	7	10 ± 1.1 a	4 ± 1.1 a	25.3 ± 0.4 a	25.6 ± 0.4 a
Trt A	Harvest 1	14	15 ± 1.8 a	12 ± 2.8 a	25.2 ± 0.3 a	25.5 ± 0.3 a
Trt B	Harvest 1	14	12.5 ± 1.8 a	11.5 ± 2.8 a	25.4 ± 0.3 a	25.7 ± 0.3 a
Trt C	Harvest 1	14	13 ± 1.8 a	9 ± 2.8 a	24.6 ± 0.3 a	24.9 ± 0.3 a
Trt A	Harvest 1	21	14.5 ± 2.5 a	11 ± 1.6 a	12.8 ± 0.4 a	12.9 ± 0.4 a
Trt B	Harvest 1	21	16.5 ± 2.5 a	10.5 ± 1.6 a	12.9 ± 0.4 a	13.1 ± 0.4 a
Trt C	Harvest 1	21	15.5 ± 2.5 a	7.5 ± 1.6 a	12.4 ± 0.4 a	12.6 ± 0.4 a
Trt A	Harvest 2	7	21.5 ± 2.7 a	11 ± 2.4 b	30 ± 0.3 a	30.3 ± 0.3 a
Trt B	Harvest 2	7	18 ± 2.7 a	8.5 ± 2.4 b	30.4 ± 0.3 a	30.8 ± 0.3 a
Trt C	Harvest 2	7	27 ± 2.7 a	24.5 ± 2.4 a	24.8 ± 0.3 b	25.1 ± 0.3 b
Trt A	Harvest 2	14	19 ± 2.2 ab	11.5 ± 1.8 ab	10.9 ± 0.3 b	11.2 ± 0.3 b
Trt B	Harvest 2	14	15 ± 2.2 b	9 ± 1.8 b	11.4 ± 0.3 ab	11.7 ± 0.3 ab
Trt C	Harvest 2	14	28.5 ± 2.2 a	16 ± 1.8 a	11.9 ± 0.3 a	12.3 ± 0.3 a
Trt A	Harvest 2	21	18.5 ± 2.8 b	10.5 ± 1.8 b	14.4 ± 0.3 a	14.6 ± 0.4 a
Trt B	Harvest 2	21	13.5 ± 2.8 b	7.5 ± 1.8 b	14.1 ± 0.3 a	14.3 ± 0.4 a
Trt C	Harvest 2	21	37.5 ± 2.8 a	20 ± 1.8 a	14.2 ± 0.3 a	14.4 ± 0.4 a
Trt A	Harvest 3	7	17.5 ± 2.5 b	14 ± 2.5 b	24 ± 0.3 a	24.2 ± 0.4 a
Trt B	Harvest 3	7	8 ± 2.5 c	6.5 ± 2.5 b	23.5 ± 0.3 a	23.9 ± 0.4 a
Trt C	Harvest 3	7	43.5 ± 2.5 a	29 ± 2.5 a	12.2 ± 0.3 b	12.3 ± 0.4 b
Trt A	Harvest 3	14	19.5 ± 3 b	13.5 ± 2.2 b	11.3 ± 0.5 c	11.6 ± 0.5 b
Trt B	Harvest 3	14	11.5 ± 3 b	6 ± 2.2 c	26.4 ± 0.5 a	26.8 ± 0.5 a
Trt C	Harvest 3	14	45.5 ± 3 a	30.5 ± 2.2 a	14.3 ± 0.5 b	14.7 ± 0.5 c
Trt A	Harvest 3	21	20 ± 2.8 b	6.5 ± 3 b	13.4 ± 0.8 a	13.6 ± 0.8 a
Trt B	Harvest 3	21	12.5 ± 2.8 b	8.5 ± 3 b	13.3 ± 0.8 a	13.6 ± 0.8 a
Trt C	Harvest 3	21	44.5 ± 2.8 a	31 ± 3 a	10.7 ± 0.8 a	10.9 ± 0.8 a

^1^Picking intervals where Trt A: 2 days. Trt B: 3 days, and Trt C: 7 days.

^2^100 berries from each replication were evaluated.

Fruit were stored at 1 °C and 85% RH. Parameters measured include berry damage (%) such as splitting, juice leakage from the pedicel, wet scar, and skin tearing, and berry diameter (mm). Values are presented as mean ± standard error (SE) for each parameter. Comparisons are made between picking intervals within each storage duration and means followed by different letters are significantly different at *p* ≤ 0.05 based (**Supplementary Table 1**) on Tukey’s honestly significant difference (HSD). Corresponding *p*-values are provided in **Supplementary Table 1**.

### 3.3 Berry diameter

Berry diameter evaluated at harvest 1 and 2 was not significantly affected by treatments in either year (**Figures 4B, D**). However, at harvest 3 of 2023 and 2024, Trt A berries had significantly smaller berry diameters compared to Trt B and C (**Figures 4B, D**). Berries from harvest 1 during storage in 2023 and 2024 did not show any significant differences (**Table 1**). After seven days of cold storage Trt A and B berries from harvests 2 and 3 were significantly larger compared to Trt C in 2023 and 2024 (**Table 1**). After 14 days of storage, the berry diameter was significantly bigger in Trt C compared to Trt A in harvest 2, while in harvest 3, Trt B berries had a bigger diameter compared to Trt A and C in 2023 and 2024 (**Table 1**). It should be noted that after 21 days of storage, there were no significant differences in berry diameter for harvests 2 and 3 in both years.

### 3.4 Firmness

**Figure 5:**
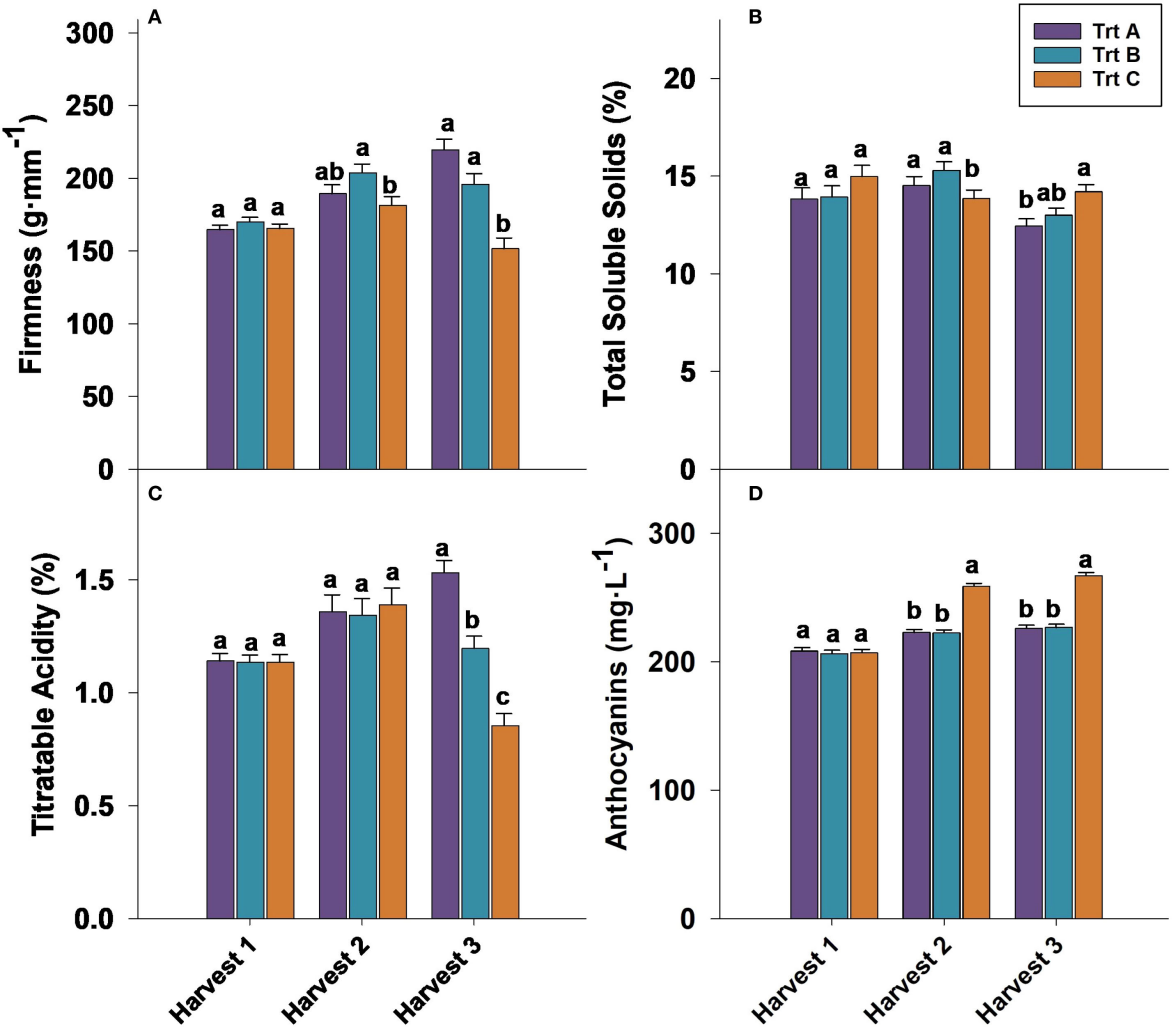
Effect of three different picking intervals on firmness **(A)** total soluble solids **(B)** titratable acidity **(C)** and anthocyanin concentration **(D)** on the ‘Brightwell’ cultivar at harvest in 2023. Comparisons were made between treatments separately for each harvest. The means followed by the different letters are significantly different at *p* ≤ 0.05 based on Tukey’s honestly significant difference (HSD). Corresponding *p*-values are provided in **Supplementary Table 1**.

**Figure 6:**
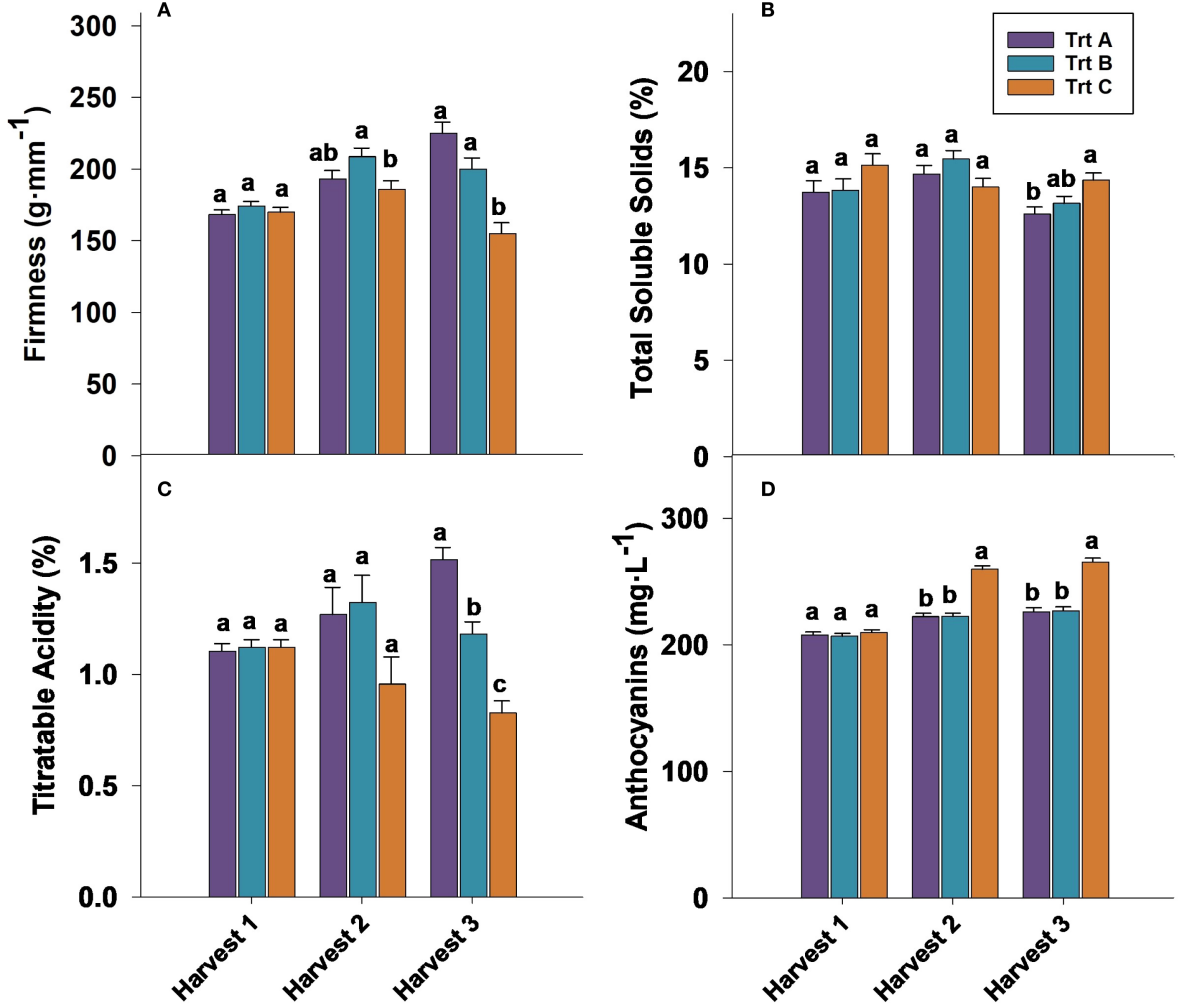
Effect of three different picking intervals on firmness **(A)**, total soluble solids **(B)**, titratable acidity **(C)**, and anthocyanin concentration **(D)** on the ‘Brightwell’ cultivar at harvest in 2024. Comparisons were made between treatments separately for each harvest. The means followed by the different letters are significantly different at *p* ≤ 0.05 based on Tukey’s honestly significant difference (HSD). Corresponding *p*-values are provided in **Supplementary Table 1**.

Firmness at harvest 1 was not significantly influenced by treatments in 2023 and 2024 (**Figures 5A**, **6A**). However, in both years analyzed, berries from Trt B collected at harvest 2 exhibited the highest firmness, while Trt C recorded the lowest firmness of 181.39 g·mm^−1^ in 2023 and 185.92 g·mm^−1^ in 2024. At harvest 3, berries of Trt A and B resulted in the highest firmness, whereas Trt C consistently showed the lowest firmness of 151.63 g·mm^−1^ in 2023 and 155.01 g·mm^−1^ in 2024 (**Figures 5A**, **6A**). After seven days of storage, berry firmness was significantly higher in Trt B berries compared to Trt C in the harvests 2 and 3, in both years analyzed (**Table 2**). Additionally, at the same harvest, firmness evaluated after 14 days of storage was significantly higher for Trt B compared to Trt A and C. In 2023 and 2024, 21 DAS, berry firmness in berries from harvest 2 was significantly higher for Trt B compared to Trt C. In harvest 3, blueberries of Trt A and B had higher firmness compared to Trt C (**Table 2**).

**Table 2:** Effects of picking intervals on fruit quality parameters of ‘Brightwell’ at each storage duration (7, 14, and 21 DAS) in 2023 and 2024. Fruit were stored at 1°C and 85% RH.

Treatment^1^	Harvest	Storage days	Firmness (g·mm^-1^)		Total soluble solids (%)		Titratable acidity^2^ (%)		Anthocyanins concentration^3^	
			2023	2024	2023	2024	2023	2024	2023	2024
Trt A	Harvest 1	7	161.4 ± 5.8 a	165.4 ± 6 a	12.6 ± 0.8 a	13 ± 0.7 b	0.8 ± 0.1 a	0.8 ± 0.1 a	208.8 ± 1.8 a	208.5 ± 1.9 a
Trt B	Harvest 1	7	167.3 ± 5.8 a	171.5 ± 6 a	15.9 ± 0.8 a	16.1 ± 0.7 a	1 ± 0.1 a	1 ± 0.1 a	207.4 ± 1.8 a	207.4 ± 1.9 a
Trt C	Harvest 1	7	154.6 ± 5.8 a	158.4 ± 6 a	14.8 ± 0.8 a	15 ± 0.7 ab	0.9 ± 0.1 a	0.9 ± 0.1 a	209.6 ± 1.8 a	210.3 ± 1.9 a
Trt A	Harvest 1	14	143.4 ± 4.1 a	147 ± 4.3 a	14.2 ± 0.5 a	14.3 ± 0.5 a	0.8 ± 0.1 a	0.8 ± 0.1 a	208.4 ± 2.2 a	208.4 ± 2.7 a
Trt B	Harvest 1	14	134.7 ± 4.1 a	138.8 ± 4.3 a	15.5 ± 0.5 a	15.7 ± 0.5 a	1 ± 0.1 a	1 ± 0.1 a	209.9 ± 2.2 a	210 ± 2.7 a
Trt C	Harvest 1	14	126.7 ± 4.1 a	129.9 ± 4.3 a	14.5 ± 0.5 a	14.6 ± 0.5 a	0.8 ± 0.1 a	0.8 ± 0.1 a	208.3 ± 2.2 a	208.1 ± 2.7 a
Trt A	Harvest 1	21	121.8 ± 4.6 a	124.8 ± 4.7 a	16.3 ± 0.7 a	15.9 ± 0.6 a	0.9 ± 0.1 a	0.8 ± 0.1 a	208.2 ± 2.3 a	208.3 ± 2.1 a
Trt B	Harvest 1	21	131.5 ± 4.6 a	134.7 ± 4.7 a	15.5 ± 0.7 a	15.6 ± 0.6 a	1.1 ± 0.1 a	1.1 ± 0.1 a	208.5 ± 2.3 a	208.4 ± 2.1 a
Trt C	Harvest 1	21	122.2 ± 4.6 a	125.3 ± 4.7 ab	15.1 ± 0.7 a	15.2 ± 0.6 a	1 ± 0.1 a	1 ± 0.1 a	204.5 ± 2.3 a	204.7 ± 2.1 a
Trt A	Harvest 2	7	169 ± 3.3 ab	172.7 ± 3.4 a	13.7 ± 0.7 a	13.8 ± 0.7 a	1.3 ± 0.2 a	1.3 ± 0.2 a	223.2 ± 1.9 b	222.9 ± 2.3 b
Trt B	Harvest 2	7	173.5 ± 3.3 a	177.7 ± 3.4 a	13.3 ± 0.7 a	13.4 ± 0.7 a	1.2 ± 0.2 a	1.2 ± 0.2 a	222.7 ± 1.9 b	222.3 ± 2.3 b
Trt C	Harvest 2	7	157.6 ± 3.3 b	161.5 ± 3.4 b	14.2 ± 0.7 a	14.1 ± 0.7 a	0.9 ± 0.2 a	0.8 ± 0.2 a	248 ± 1.9 a	259.6 ± 2.3 a
Trt A	Harvest 2	14	159.1 ± 4.3 b	163 ± 4.4 b	14.8 ± 0.4 b	15 ± 0.4 ab	1.3 ± 0.1 ab	1.3 ± 0.1 a	222.2 ± 2 b	222.2 ± 1.3 b
Trt B	Harvest 2	14	176.8 ± 4.3 a	181.3 ± 4.4 a	15.7 ± 0.4 a	15.7 ± 0.4 a	1.4 ± 0.1 a	1.4 ± 0.1 a	223.8 ± 2 b	223.8 ± 1.3 b
Trt C	Harvest 2	14	147.1 ± 4.3 b	150.5 ± 4.4 b	14.1 ± 0.4 b	14.2 ± 0.4 b	1.1 ± 0.1 b	1 ± 0.1 b	249.5 ± 2 a	269.4 ± 1.3 a
Trt A	Harvest 2	21	164.5 ± 5.9 ab	171.3 ± 6.5 ab	15.5 ± 0.4 a	15.7 ± 0.4 a	1.2 ± 0.1 a	1.1 ± 0.1 a	221.4 ± 1.7 b	221.6 ± 1.5 b
Trt B	Harvest 2	21	191.3 ± 5.9 a	195.6 ± 6.5 a	15 ± 0.4 a	15.2 ± 0.4 a	1.2 ± 0.1 a	1.2 ± 0.1 a	222.3 ± 1.7 b	222 ± 1.5 b
Trt C	Harvest 2	21	150.6 ± 5.9 b	154.3 ± 6.5 b	14.7 ± 0.4 a	14.9 ± 0.4 a	1.2 ± 0.1 a	1.1 ± 0.1 a	253.4 ± 1.7 a	261.6 ± 1.5 a
Trt A	Harvest 3	7	196.1 ± 5.7 a	200.2 ± 6.2 a	14 ± 0.4 a	14.5 ± 0.3 a	1.7 ± 0.1 a	1.7 ± 0.1 a	224.7 ± 3 b	224.6 ± 3.5 b
Trt B	Harvest 3	7	191.1 ± 5.8 a	193.2 ± 6.2 a	14 ± 0.4 a	14.2 ± 0.3 a	1.3 ± 0.1 ab	1.3 ± 0.1 b	225.3 ± 3 b	225.6 ± 3.5 b
Trt C	Harvest 3	7	145.3 ± 5.7 b	147.7 ± 6.2 b	12.4 ± 0.4 b	12.3 ± 0.3 b	1 ± 0.1 b	1 ± 0.1 b	268.9 ± 3 a	262.2 ± 3.5 a
Trt A	Harvest 3	14	168.9 ± 5 b	173 ± 5.2 b	16.5 ± 0.5 a	16.7 ± 0.5 a	1.4 ± 0.1 a	1.4 ± 0.1 a	225 ± 3.1 b	224.9 ± 2.9 b
Trt B	Harvest 3	14	193.1 ± 4.9 a	197.7 ± 5.2 a	14.1 ± 0.5 b	14.3 ± 0.5 b	1.4 ± 0.1 a	1.4 ± 0.1 a	224.1 ± 3.1 b	223.9 ± 2.9 b
Trt C	Harvest 3	14	146.8 ± 5 c	148.7 ± 5.2 c	12.9 ± 0.5 b	12.8 ± 0.5 b	1 ± 0.1 b	1 ± 0.1 b	257.9 ± 3.1 a	266 ± 2.9 a
Trt A	Harvest 3	21	186.1 ± 5 a	190.8 ± 4.9 a	16.5 ± 0.7 a	16.5 ± 0.8 a	1.7 ± 0.1 a	1.7 ± 0.1 a	224.9 ± 3.9 b	224.5 ± 2.3 b
Trt B	Harvest 3	21	181.6 ± 5.1 a	184.3 ± 4.9 a	14.9 ± 0.7 b	15.1 ± 0.8 a	1.4 ± 0.1 b	1.4 ± 0.1 a	227.4 ± 3.9 b	227.7 ± 2.3 b
Trt C	Harvest 3	21	132.3 ± 5 b	135.3 ± 4.9 b	13.5 ± 0.7 b	13.7 ± 0.8 b	0.9 ± 0.1 c	1 ± 0.1 b	262.8 ± 3.9 a	263.7 ± 2.3 a

^1^Picking intervals where Trt A: 2 days. Trt B: 3 days, and Trt C: 7 days.

^2^Titratable acidity (TA) is expressed as percent citric acid.

^3^Anthocyanin concentration in mg·L^-1^ cyanidin-3-glucoside.

Parameters measured include firmness (g·mm^−1^), total soluble solids (%), titratable acidity (%), and anthocyanin concentration (mg·L^−1^). Values are presented as mean ± standard error (SE) for each parameter. Comparisons are made between picking intervals within each storage duration and means followed by different letters are significantly different at *p* ≤ 0.05 based on Tukey’s honestly significant difference (HSD). Corresponding *p*-values are provided in **Supplementary Table 1**.

### 3.5 Total soluble solids (%)

The TSS assessed at harvest 1 did not exhibit significant differences among the treatments in 2023 and 2024. However, in 2023, Trt A and B berries showed higher TSS levels of 15% and 15.3% at harvest 2 compared to Trt C, whereas no significant differences in TSS were observed at harvest 2 in 2024 (**Figures 5B**, **6B**). It should be noted that at harvest 3, berries from Trt C showed significantly higher TSS levels. Specifically, in 2023, TSS levels were 14% for Trt C, compared to 12.5% and 13.0% for Trt A and B, respectively. A similar trend was observed in 2024, with Trt C recording a TSS of 14%, while Trt A and B had TSS levels of 13% and 13.2%, respectively. (**Figures 5B**, **6B**).

Berries from harvest 1 did not show significant differences in TSS levels at different storage dates (**Table 2**). In 2023 and 2024, TSS after seven days of storage was not significantly affected by treatments in berries from harvest 2; however, in harvest 3, Trt A berries had significantly higher TSS compared to Trt C berries (**Table 2**). After 14 days of storage in both years evaluated, Trt B berries exhibited the highest TSS compared to Trt C in harvest 2, while for harvest 3, Trt A berries had higher TSS compared to Trt B and C (**Table 2**). After 21 days of storage in 2023 and 2024, no significant differences in TSS in harvest 2 were observed among the treatments, but in harvest 3, Trt C berries showed significantly lower TSS compared to Trt A and B in both years (**Table 2**).

### 3.6 Titratable acidity

Titratable acidity (TA) at harvests 1 and 2 was not significantly affected by treatments in 2023 and 2024. However, at harvest 3 berries of Trt A showed significantly higher TA of 1.53% and 1.51% compared to Trt B and C (**Figures 5C**, **6C**). During the storage period, the TA of blueberries remained relatively stable across the harvests, but between treatments, significant differences were observed. For instance, the TA of berries from harvest 2 after seven days of storage was not significantly different, but in harvest 3, Trt A berries had significantly higher TA compared to Trt C during both years (**Table 2**). After 14 days of storage, the TA of berries was significantly higher for Trt A and B compared to Trt C in harvests 2 and 3 in 2024. After 21 days of storage, no significant differences in TA were observed in harvest 2 between 2023 and 2024. However, in harvest 3, Trt A and B maintained higher TA levels than Trt C in both years (**Table 2**).

### 3.7 Anthocyanins concentration

In both 2023 and 2024, anthocyanin concentration was significantly higher in blueberries in Trt C compared to Trt A and B at harvests 2 and 3 (**Figures 5D**, **6D**). Specifically, in 2023, anthocyanin levels in Trt C were 258.89 mg·L^−1^ at harvest 2 and 267.19 mg·L^−1^ at harvest 3. Similarly, in 2024, anthocyanin levels in Trt C were 259.84 mg·L^−1^ at harvest 2 and 265.42 mg·L^−1^ at harvest 3. This trend continued through 7, 14, and 21 DAS, with the berries from Trt C consistently showing the highest anthocyanin concentration across harvests 2 and 3 in both years (**Table 2**).

## 4 Discussion

The results of this study demonstrated that picking intervals have a significant impact on the postharvest quality and storability of rabbiteye blueberries cv. ‘Brightwell.’ The increase in weight loss observed in Trt C, with longer picking intervals, across both years, suggests that extended periods between harvests negatively affected quality during the 21-day storage period. The advanced ripeness stage in Trt C likely makes these berries more susceptible to dehydration. As fruit ripens, cuticle thickness and cuticle wax content decrease, leading to a higher water permeability (Yan and Castellarin, 2022; Yan et al., 2024). Previous studies indicate that blueberries are considered unmarketable once weight loss exceeds 5 to 8% (Sanford et al., 1991), with other research suggesting that the acceptable limit during a 14 to 21-day storage period ranges between 5% and 7% (Paniagua et al., 2014). More frequent harvests, as seen in Trt A and B, helped mitigate the weight loss issue by ensuring berries are collected at an optimal ripeness stage, thus reducing postharvest weight loss.

Berries from Trt C exhibited lower firmness and higher berry damage, which can be explained by internal structure changes that occur during ripening and senescence. Blueberries undergo a softening process driven by the enzymatic breakdown of cell wall components, including pectin, cellulose, and hemicellulose (Chen et al., 2015; Proctor and Miesle, 1991; Silva et al., 2005). Weakening cell walls can make fruit more prone to softening and internal damage (Chen et al., 2015; Silva et al., 2005). Thus, accumulation of soft and overripe fruit could lead to increased softening incidence, damage, and decay during storage, resulting in lower firmness and poor overall quality (Lobos et al., 2018; Moggia et al., 2017b; Strik, 2019). Additionally, Moggia et al. (2017a), reported that factors such as stem scar or berry damage can also increase water loss and reduce firmness in blueberries during storage. Firmness is crucial for marketability, as firmer berries are less prone to mechanical damage and decay during postharvest handling (Vicente et al., 2007). Our research shows that lower berry damage rates in blueberries from Trt A and B highlight how frequent harvesting helps maintain postharvest fruit quality. These outcomes emphasize the importance of minimizing weight loss during storage by using shorter picking intervals to maintain postharvest fruit quality. These findings are consistent with those of Miller et al. (1988); Chen (2006); Lobos et al. (2018), and Moggia et al. (2022), who reported higher postharvest damage susceptibility in blueberries harvested at weekly intervals due to the presence of overripe berries, which are more prone to decay compared to ripe or immature berries. Recent work from our group in southern highbush and rabbiteye blueberries in Georgia confirmed that delaying harvests by one or two weeks negatively impacts quality at harvest and during storage (Godara et al., 2025).

Furthermore, Lobos et al. (2018), reported that six-day picking intervals reduce firmness by increasing the proportion of overripe fruit in northern highbush blueberries. According to Moggia et al. (2017b), fruit that remains on the bush after maturity tends to be softer at harvest and during storage, which was also confirmed by a similar trend in the present work. Strik (2019), reported that harvesting frequencies of 8 and 12 days resulted in lower firmness in northern highbush fruit. Similarly, the decline in firmness observed in fruit harvested every seven days is the result of the accumulation of overripe fruit (Lobos et al., 2018; Moggia et al., 2018, 2022; Strik, 2019).

In fruits, total soluble solids (TSS) and titratable acidity (TA) are the primary determinants of flavor, which change during fruit ripening (Zhang et al., 2020). In this work, the increase in TSS and decline in TA in blueberries across all treatments during ripening and storage was consistent as soluble solids continued to accumulate and acids were metabolized and declined as blueberries ripened, an effect that has been previously reported by several authors (Lin et al., 2020; Lobos et al., 2018; Moggia et al., 2018; Sargent et al., 2006; Shi et al., 2023; Strik, 2019). The relatively higher TSS values in blueberries from Trt C during harvest 3 can be attributed to their advanced ripening stage (Lobos et al., 2018; Strik, 2019). Lobos et al. (2018) found that fruit harvested six days after full maturity was high in TSS and low in TA compared to fruit harvested at 100% blue stage. The overripe fruit exhibited a more dramatic decline in TSS during a 45-day cold storage period. Similarly, in the present study, TSS was higher in blueberries harvested from Trt C at harvest 3, and it increased during storage of 21 days, but remained significantly lower than Trt A. In addition to the advanced stage of berry maturity, higher TSS values observed in Trt C may also be partially attributed to elevated temperatures during the second week of June, when the second and third harvests for this treatment were conducted. While Trt A and Trt B harvests occurred primarily during the first week of June, when average daily temperatures were around 23 °C (2023) and 24 °C (2024), the later harvests in Trt C coincided with higher temperatures, 26 °C in 2023 and 27 °C in 2024 (**Figures 1A, B**). These warmer conditions may have promoted more rapid sugar accumulation in the fruit, enhancing TSS alongside the effect of extended ripening time. Anthocyanins are responsible for the blue pigmentation of blueberries and consistently increase during fruit ripening (Zifkin et al., 2012). The higher anthocyanin levels in Trt C can be attributed to the longer ripening period before harvest, which allows for greater pigment accumulation (Kalt et al., 2003). As blueberries ripen, anthocyanin accumulation increases alongside sugars, reaching peak concentration at stage eight, indicating full pigment development (Acharya et al., 2024). In the present work, the anthocyanin concentration was significantly higher in berries from Trt C, which confirms the natural progression of anthocyanin biosynthesis. This highlights the importance of balancing anthocyanin content with other quality attributes like firmness, berry weight, and susceptibility to decay, which can be effectively achieved by optimizing picking intervals. While Trt C berries accumulated higher anthocyanin concentrations, this came at the cost of lower textural quality. This trade-off suggests that such fruit, although less ideal for fresh markets, may be better suited for processing applications such as juices, jams, or purees, where pigment content is valued over textural quality (Olmstead and Finn, 2014; Strik and Yarborough, 2005).

## 5 Conclusions

In conclusion, optimizing picking intervals is critical for maintaining the postharvest quality of rabbiteye blueberries intended for the fresh market. This study highlights the importance of frequent, timely harvesting, especially in warm, humid climates like Georgia, where high temperatures and precipitation can impact fruit ripening and postharvest physiology. A moderate picking interval of three days, as seen in Trt B, helps maintain postharvest quality by minimizing weight loss and reducing damage, while maintaining optimal firmness, TSS, and TA levels. In contrast, longer picking intervals (e.g., seven days) may lead to increased anthocyanin concentrations but this comes at the expense of firmness and higher postharvest damage incidence. Based on the balance between postharvest quality, weight loss, firmness, and flavor attributes, the 3-day picking interval (Trt B) is recommended as the optimal hand-picking strategy for maintaining marketability of fresh market rabbiteye blueberries. However, the choice of interval may be market-dependent, with a 3-day interval being optimal for fresh market berries, where fruit texture is prioritized, while a 7-day interval could be viable for the processing market despite the lower texture quality.

An alternative approach to reduce the labor-intensive and costly nature of multiple harvests could involve different strategies to achieve more synchronized ripening. The ripening asynchrony of blueberries could be alleviated by utilizing appropriate preharvest management practices as well as breeding efforts that can result in a more concentrated ripening fashion, diminishing the need for multiple, frequent harvests. This study was conducted on a single rabbiteye blueberry cultivar (‘Brightwell’) grown under Georgia conditions, and further research is needed to validate these findings across additional cultivars and production regions. Future research should also explore strategies to complement optimal picking interval recommendations and further enhance efficiency in blueberry production.

## Data Availability

The datasets presented in this study can be found in online repositories. The names of the repository/repositories and accession number(s) can be found below: https://doi.org/10.7910/DVN/3HQKTH.
