# Supplementary material for: Impact of shorter picking intervals on the storability and postharvest quality of rabbiteye blueberries cv. ‘Brightwell’
Source: Front Plant Sci. 2025 Oct 17;16:1683940. doi: 10.3389/fpls.2025.1683940 (PMC12575388; doi:10.3389/fpls.2025.1683940)
Supplement: Supplementary file 1 [file Table1.docx]

Supplementary Table 1: *P*-values from ANOVA showing the effect of different picking intervals on fruit quality parameters of 'Brightwell' blueberries at harvest (0 day) and after each storage duration (7, 14, and 21 days after storage, DAS). Parameters assessed include firmness (g·mm⁻¹), berry diameter (mm), total soluble solids (%), titratable acidity (%), anthocyanin concentration (mg L⁻¹), berry damage (%), and moisture loss (%). Analyses were conducted separately for each of the three harvests in 2023 and 2024. Asterisks (*) indicate statistically significant differences (p < 0.05) across storage intervals.

| Variable | Year | Storage Day | Harvest 1 | Harvest 2 | Harvest 3 |
| --- | --- | --- | --- | --- | --- |
|  |  |  | *P* value | *P* value | *P* value |
| Firmness (g·mm-1) | 2023 | 0 | 0.2805 | 0.0429* | 0.0020* |
|  | 2023 | 7 | 0.4049 | 0.0241* | 0.0001* |
|  | 2023 | 14 | 0.1092 | 0.0026* | 0.0009* |
|  | 2023 | 21 | 0.4061 | 0.0123* | 0.0001* |
|  | 2024 | 0 | 0.2302 | 0.0463* | 0.0020* |
|  | 2024 | 7 | 0.405 | 0.0249* | 0.0005* |
|  | 2024 | 14 | 0.1259 | 0.0028* | 0.0003* |
|  | 2024 | 21 | 0.406 | 0.0175* | <0.0001* |
| Berry diameter (mm) | 2023 | 0 | 0.8216 | 0.0911 | 0.0136* |
|  | 2023 | 7 | 0.5039 | <0.0001* | <0.0001* |
|  | 2023 | 14 | 0.1211 | 0.0040* | <0.0001* |
|  | 2023 | 21 | 0.5247 | 0.7518 | 0.0966 |
|  | 2024 | 0 | 0.8839 | 0.1165 | 0.0084* |
|  | 2024 | 7 | 0.5031 | <0.0001* | <0.0001* |
|  | 2024 | 14 | 0.1261 | 0.0109* | <0.0001* |
|  | 2024 | 21 | 0.5241 | 0.8652 | 0.0884 |
| Total soluble solids (%) | 2023 | 0 | 0.3399 | 0.2326 | 0.0209* |
|  | 2023 | 7 | 0.0764 | 0.4203 | 0.0038* |
|  | 2023 | 14 | 0.1525 | 0.0019* | 0.0055* |
|  | 2023 | 21 | 0.3479 | 0.3916 | 0.0091* |
|  | 2024 | 0 | 0.279 | 0.2326 | 0.0209* |
|  | 2024 | 7 | 0.0631 | 0.6975 | 0.0043* |
|  | 2024 | 14 | 0.1403 | 0.0339* | 0.0050* |
|  | 2024 | 21 | 0.7481 | 0.3916 | 0.0240* |
| Titratable acidity (%) | 2023 | 0 | 0.9892 | 0.9039 | 0.0002* |
|  | 2023 | 7 | 0.1583 | 0.1596 | 0.0032* |
|  | 2023 | 14 | 0.1653 | 0.0242* | <0.0001* |
|  | 2023 | 21 | 0.1649 | 0.4642 | 0.0007* |
|  | 2024 | 0 | 0.8905 | 0.2261 | 0.0002* |
|  | 2024 | 7 | 0.154 | 0.1514 | 0.0034* |
|  | 2024 | 14 | 0.1695 | 0.0203* | 0.0006* |
|  | 2024 | 21 | 0.1664 | 0.4441 | 0.0013* |
| Anthocyanins concentration (mg L⁻¹) | 2023 | 0 | 0.8776 | <0.0001* | <0.0001* |
|  | 2023 | 7 | 0.7221 | 0.0002* | <0.0001* |
|  | 2023 | 14 | 0.8001 | 0.0002* | 0.0004* |
|  | 2023 | 21 | 0.5155 | <0.0001* | 0.0006* |
|  | 2024 | 0 | 0.6763 | <0.0001* | 0.0002* |
|  | 2024 | 7 | 0.6606 | <0.0001* | 0.0005* |
|  | 2024 | 14 | 0.8858 | <0.0001* | <0.0001* |
|  | 2024 | 21 | 0.4849 | <0.0001* | <0.0001* |
| Berry damage (%) | 2023 | 0 | 0.4472 | 0.0011* | <0.0001* |
|  | 2023 | 7 | 0.1847 | 0.2036 | <0.0001* |
|  | 2023 | 14 | 0.493 | 0.0147* | 0.0010* |
|  | 2023 | 21 | 0.7814 | 0.0048* | 0.0005* |
|  | 2024 | 0 | 0.824 | 0.0353* | 0.0018* |
|  | 2024 | 7 | 0.6232 | 0.0081* | 0.0018* |
|  | 2024 | 14 | 0.5045 | 0.0309* | 0.0003* |
|  | 2024 | 21 | 0.2928 | 0.115* | 0.0015* |
| Moisture loss (%) | 2023 | | 0.2977 | 0.0104* | <0.0001* |
|  | 2024 | | 0.577 | <0.0001* | 0.0002* |
